# Supplementary material for: Low ocean-floor rises regulate subpolar sea surface temperature by forming baroclinic jets
Source: Nat Commun. 2018 Mar 22;9:1190. doi: 10.1038/s41467-018-03526-z (PMC5864925; doi:10.1038/s41467-018-03526-z)
Supplement: Supplementary file 1 — Supplementary Information(PDF 1779 kb) [file 41467_2018_3526_MOESM1_ESM.pdf]

Supplementary Information

Low ocean-floor rises regulate subpolar sea surface temperature by forming baroclinic jets

Humio Mitsudera

Supplementary Figure 1: IJI time series vs OEI time series derived from OISST

Supplementary Figure 2: SST, heat advection and air-sea heat flux variations with respect to the jet strength changes

Supplementary Figure 3: Blocking of westward propagation of SSH signals at the Isoguchi J1

Supplementary Figure 4: Schematic plot of the Isoguchi J1 formation

Supplementary Figure 5: Surface intensification of J1 and J2

Supplementary Reference

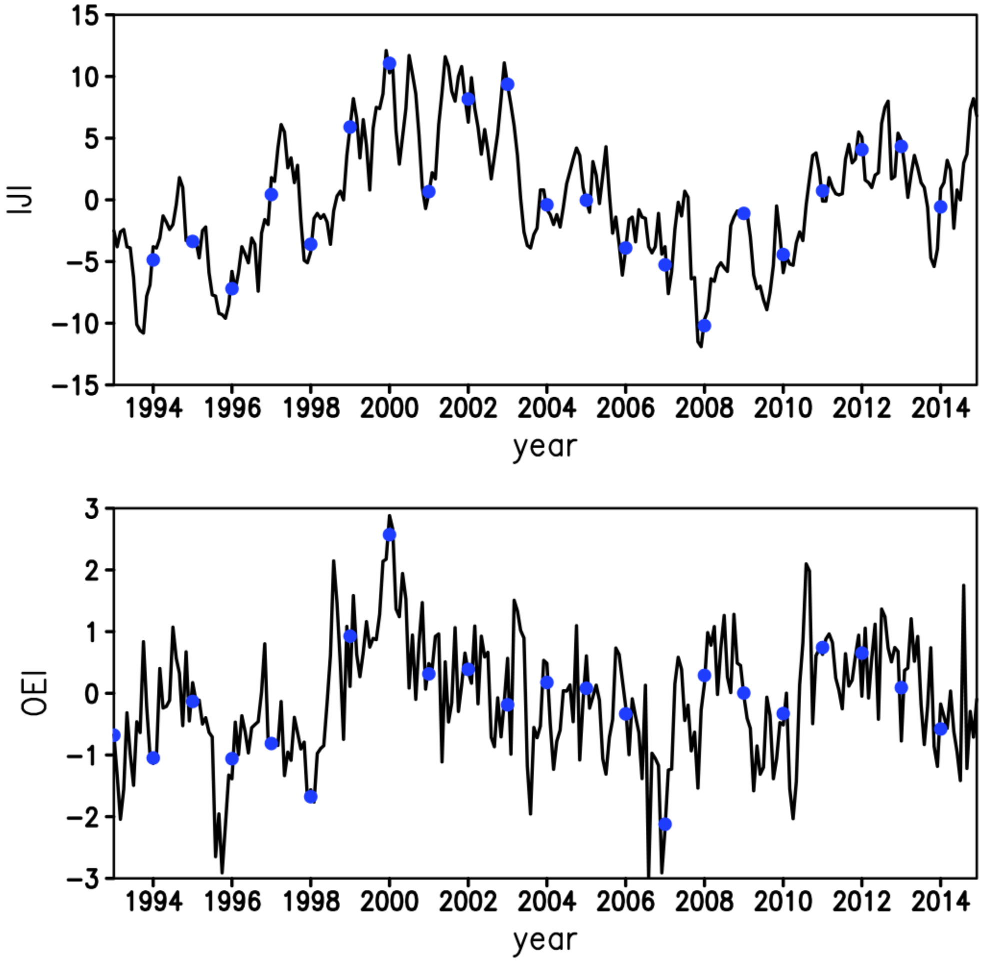

Supplementary Figure 1 IJI time series vs OEI time series derived from OISST [Upper panel] Monthly Isoguchi Jet Index (IJI) time series defined by the SSH difference between the boxes indicated in Fig. 2a in the main text. [Lower panel] The Oyashio Extension Index (OEI) time series derived from the OISST, and their wintertime (December, January, February) values (blue dots). The OEI is the same as that of Frankignoul et al.[1] except that the time series is extended to 2015, although the time series is shown from 1993. As written in the main text, the IJI time series is correlated significantly with the OEI time series, where the correlation coefficient is 0.64 with respect to the wintertime time series with the significance level greater than 95 %.

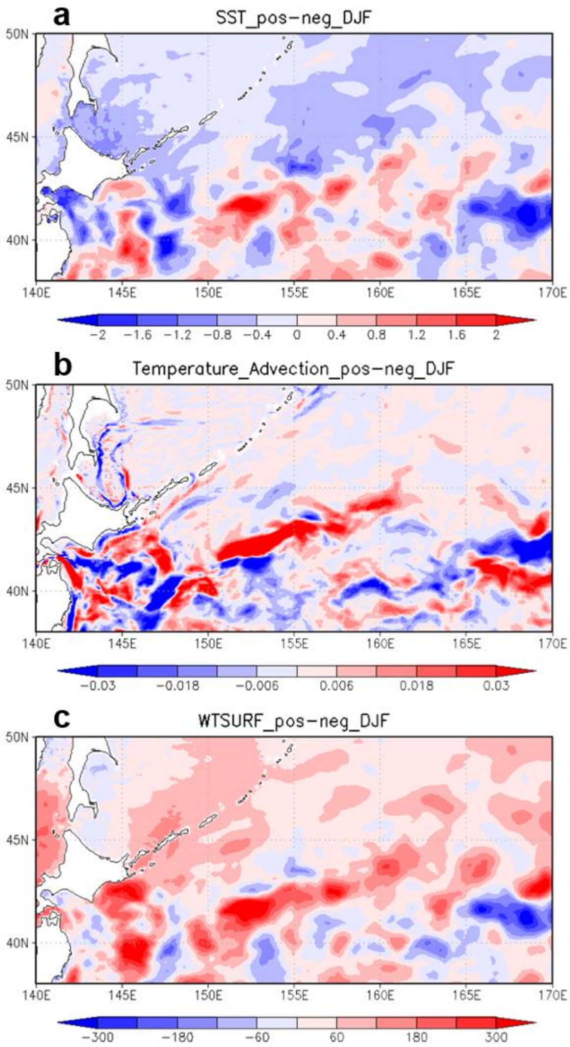

Supplementary Figure 2 SST, heat advection and air-sea heat flux variations with respect to the jet strength changes (a) Wintertime (December, January, February; DJF) SST difference between a large-IJI-year composite (1999, 2000, 2002, 2003) and a small-IJI-year composite (1996, 1998, 2006, 2007, 2008) derived from JCOPE2. The strongest warming is seen around 42°N, 153°E when the IJI increases, which coincides with the location of the largest regression coefficient in Fig. 2a of the main text. Further, the warming around 42.5°N, 157°E and that around 43°N, 160°E are also collocated with the regions of significant correlation between the SST and the IJI (Fig. 2a). (b) Difference in the linearized heat advection term $\boldsymbol{v}' \cdot \nabla\bar{T} + \bar{\boldsymbol{v}} \cdot \nabla T'$ (°C day^{-1}) during DJF between the above two composites, where $\boldsymbol{v}, T$ denote the surface velocity vector and SST, respectively, prime denotes the difference between the two composites, and bar denotes the mean. The difference in the heat advection term is large over the J1 when the J1 is strong. This implies that SST over the J1 is warmed because of the increased heat adivection. (c) Difference in the DJF air-sea heat flux (W m^{-2}) between the above two composites. Positive denotes upward. Heat flux of 100 W m^{-2} cools water column of a thickness of 200 m by approximately 0.01 °C per day, where 200 m is a typical mixed layer depth in the SAFZ[2]. The difference in the upward air-sea heat flux is large where the J1 flows, whose value is in the same order as (but smaller than) the heat advection term in (b). This is consistent with Smirnov et al.[3].

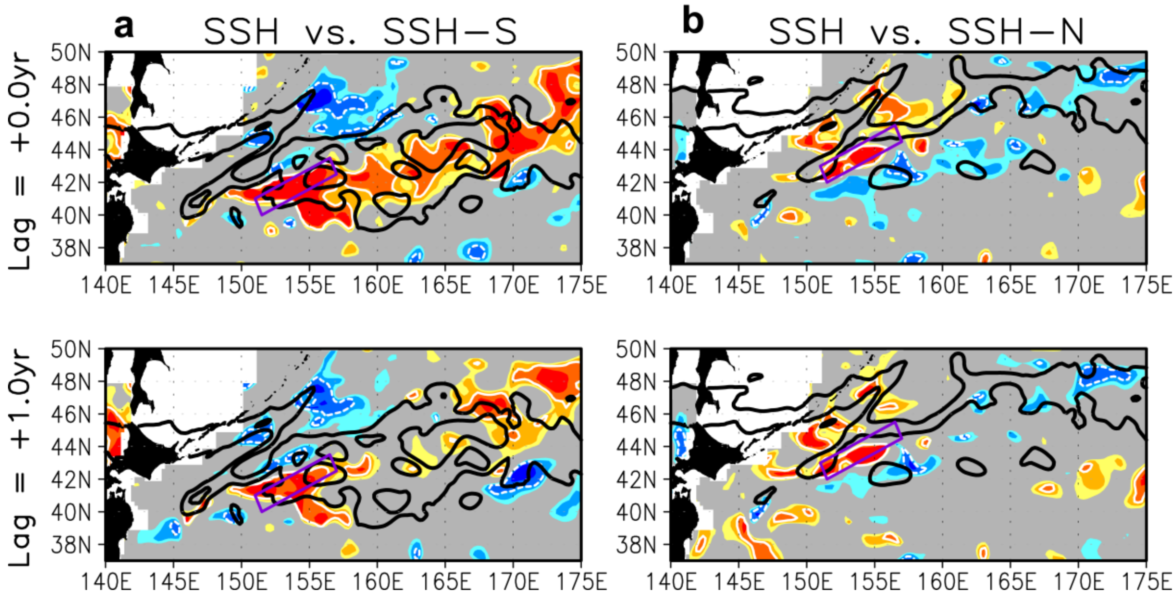

Supplementary Figure 3 Blocking of westward propagation of SSH signals at the Isoguchi J1 (a) Lag correlation map of SSHs with respect to the SSH-S. Lower panel displays SSH signals with the 1-year lag (SSH-S leads the SSHs). Upper panel display simultaneous correlation (0-year lag) for reference. The SSH signals in the southeastern box at the lag 0 year does not propagate westward but almost stationary inside the box. (b) Same as (a) but for the correlation with respected to the SSH-N.

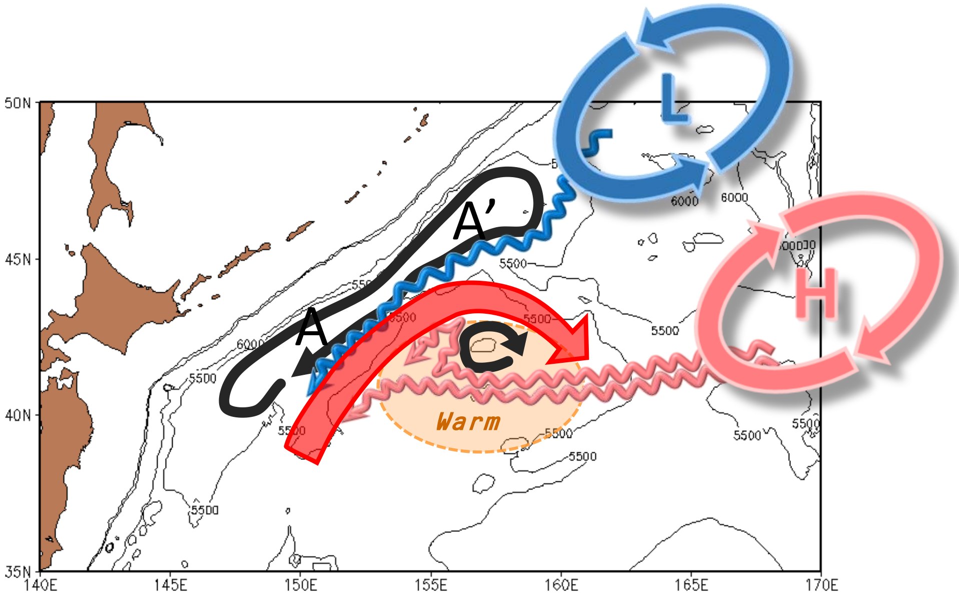

Supplementary Figure 4 Schematic plot representing the formation of the Isoguchi J1 (red thick arrow) Undulating pink (blue) arrows denote baroclinic Rossby waves of subtropical (subarctic) origin that bring thick (thin) upper layer. Thick black arrows denote barotropic recirculation over the low bottom rises focused in this study. The contour lines of a depth of 5500 m are indicated. Elliptic circle with the pink (blue) color represents anticyclonic (cyclonic) wind stress curl.

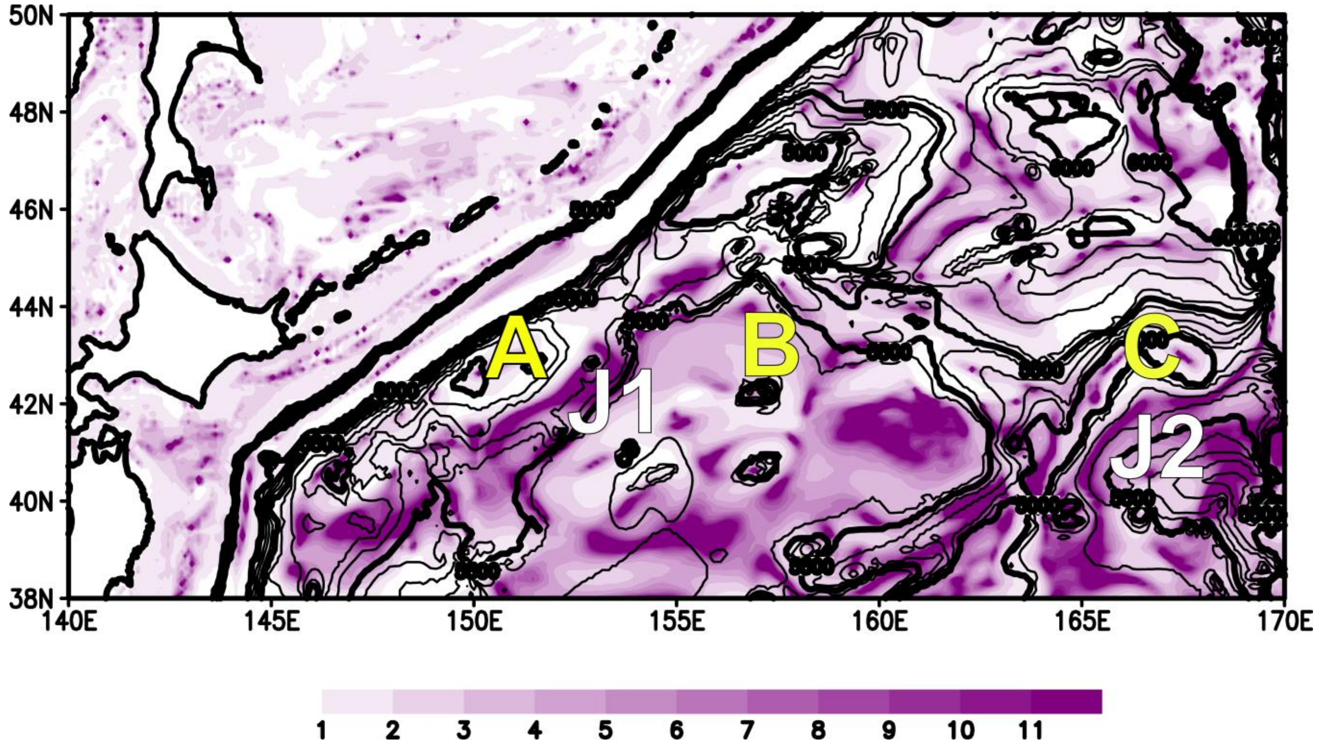

Supplementary Figure 5 Surface intensification of the J1 and the J2. Shade indicates the ratio between the surface current speed of a depth of 10 m and the barotropic flow speed. Bottom topographic contour between 5000 m and 6000 m are indicated with an interval of 100 m. The surface current speed of the J1 is intensified by a factor of 10 between 40 °N, 150 °E and 43 °N, 153 °E on the eastern flank of the rise A. The surface intensification reduces eastward of 43 °N, 153 °E, where the sea floor is relatively flat. In this downstream region, the J1 rides on the barotropic recirculation around the rise B (Fig. 1b in the main text). Surface current speed of the J2 is surface intensified by a factor of more than 10 all the way between 40 °N, 165 °E and 43 °N, 170 °E over the eastern flank of the rise C.

Supplementary Reference

1. Frankignoul, C., Senne'chael, N., Y. Kwon, M. A. Alexander Influence of the Meridional Shifts of the Kuroshio and the Oyashio Extensions on the Atmospheric Circulation. *J. Clim.*, **24,** 762-777, DOI: 10.1175/2010JCLI3731.1 (2011).

2. Suga, T., K. Motoki, Y. Aoki, and A. M. Macdonald, The North Pacific climatology of winter mixed layer and mode waters, *J. Phys. Oceanogr.,* **34,** 3 – 22 (2004).

3. Smirnov, D., M. Newman, and M.A. Alexander, Investigating the role of ocean–atmosphere coupling in the North Pacific Ocean. *J. Clim.,* **27,** 592–606.doi:10.1175/JCLI-D-13-00123.1 (2014).
